# Estimating the Frequency of Inpatient Adverse Events Using a 2-Step Retrospective Chart Review: Initial Observational Cohort Study

**DOI:** 10.2196/82512

**Published:** 2025-12-29

**Authors:** Syed Sabih Ul Hassan, Fozia Asif, Farwa Ayub, Ghazal Haque, Asad Latif

**Affiliations:** 1 Aga Khan University Karachi, Sindh Pakistan

**Keywords:** patient safety, patient harm, adverse events, inpatients, retrospective studies, low- and middle-income country

## Abstract

**Background:**

Lower- and middle-income countries bear a disproportionate share of the global burden of adverse events in health care. Despite this, patient safety research is predominantly conducted in high-income countries with well-developed health care systems, resulting in evidence and methodologies that have limited applicability in resource-constrained settings.

**Objective:**

This pilot study primarily aimed to identify the most suitable methodology for a full-scale study to detect inpatient adverse events at a tertiary care hospital in a lower- to middle-income country. Second, we aimed to use our experience with this study to further adapt the selected methodology to our setting.

**Methods:**

This external pilot study used a 2-step retrospective chart review methodology. Two separate screening tools, tools 1A and 1B, were compared to identify which performed better in our setting. We reviewed the medical records of patients who were discharged between January 1, 2019, and December 31, 2019, from a tertiary care hospital in a lower- to middle-income country in South Asia. The main outcome of interest was the rate of adverse events among hospitalized patients reported as the total number of adverse events experienced per 100 admissions.

**Results:**

A total of 100 medical records were screened using tool 1A, with the mean patient age being 39.2 (SD 27.7) years and the mean length of stay being 3.3 (SD 2.8) days. Only 1 adverse event was identified using tool 1A, resulting in an adverse event rate of 1 event per 100 hospital admissions. Tool 1B was also used to screen a total of 100 medical records. The mean patient age was found to be 39.8 (SD 28.4) years, with the mean length of stay being 3.5 (SD 3.4) days. A total of 30 adverse events were identified across 22 patient files, with 18 (60%) considered preventable, resulting in an adverse event rate of 30 events per 100 hospital admissions.

**Conclusions:**

This study demonstrates that tool 1B, adapted from the Global Trigger Tool for Measuring Adverse Events, represents an appropriate and sensitive methodology to identify adverse events among hospitalized patients in a lower- to middle-income country. Furthermore, the findings and experiences of this study were used to improve the design and procedures of our research methodology before implementation in a full-scale study.

**International Registered Report Identifier (IRRID):**

RR2-10.1136/bmjopen-2023-076971

## Introduction

Unsafe patient care during health care delivery is one of the 10 leading causes of death and disability worldwide [1]. Worldwide, the frequency of adverse events in hospitalized patients has been estimated to range between 2.9% and 21.9%, with approximately half of these events considered to be preventable [2]. The most frequent patient safety events encountered in hospitalized patients include medication errors; health care–associated infections; unsafe surgical procedures, injections, and blood transfusions; venous thromboembolism; and diagnostic and radiation errors [3]. In the United States alone, medical errors result in health care costs of approximately US $20 billion and contribute to more than 250,000 deaths annually [4,5]. It is estimated that the global economic cost of unsafe care exceeds US $1 trillion annually [6]. However, the burden of unsafe care disproportionately affects low- and middle-income countries (LMICs), where it is responsible for 134 million adverse events and 2.6 million deaths annually [7]. In LMICs, poor-quality care now represents a greater barrier to reducing mortality than limited access to care, with approximately 60% of deaths associated with treatable conditions now resulting from deficiencies in the quality of care delivered [8].

Despite this long-standing disparity in burden, the vast majority of patient safety research has originated from high-income countries (HICs), underscoring a broader global trend in which scientific knowledge production is dominated by a small number of high-income nations [9,10]. However, in recent years, global awareness of the need to enhance patient safety standards has resulted in a modest increase in patient safety research in LMICs [11,12]. It is now widely recognized that local researchers are better able to establish health research priorities that are relevant to their populations; design contextually appropriate interventions; and effectively bridge the gap among evidence, policy, and implementation [13,14]. Therefore, a crucial first step in addressing unsafe care in LMICs is to generate locally derived evidence by identifying adverse events within health care settings, thereby determining the true burden and nature of the problem.

Worldwide, a range of methodologies and tools have been developed to detect health care–associated harm [15]. However, keeping resource constraints in LMICs in mind, it is important to use an adverse event detection methodology that is both accurate and feasible. Historically, most studies concerned with adverse events have used various medical record review methodologies that have greater sensitivity than other methods, including voluntary reporting [16,17]. The 2 most popular tools, derived from the Harvard Medical Practice Study and the Global Trigger Tool for Measuring Adverse Events, have been repeatedly validated and compared in studies conducted in HICs [2]. However, it is expected that, for a number of reasons, including rudimentary medical record documentation systems and a lack of available expertise, the results of those studies are not applicable to LMICs [7]. Furthermore, to the best of our knowledge, there are no real-world data comparing the 2 aforementioned tools exclusively from LMICs. Therefore, the primary aim of this external pilot study was to assess which of the 2 methodologies is most suitable for detecting adverse events in a tertiary health care setting in an LMIC. A secondary aim was to draw on the experience gained during the pilot to refine the selected methodology, including improving the data collection tools and workflow processes, in preparation for a larger study that will measure the incidence of adverse events in hospitalized patients.

## Methods

### Study Design

This pilot study used an observational cohort design where the medical records of patients discharged between January 1, 2019, and December 31, 2019, were randomized and retrospectively reviewed to identify adverse events experienced by inpatients. We piloted 2 separate trigger lists to allow us to select the most appropriate methodology for our larger study. Our published protocol provides more details regarding our larger study [18].

### Study Setting

The study site is a 710-bed private, tertiary care teaching hospital that is located in an LMIC in South Asia and provides care to over 50,000 inpatients and 900,000 outpatients annually from all over the country. The hospital is organized into 11 clinical service lines: (1) heart, lung, and vascular; (2) kidney and bladder; (3) mind and brain; (4) cancer care; (5) internal medicine; (6) eye and ear, nose, and throat; (7) gastrointestinal and surgery; (8) anesthesiology, operating room, and central sterile supply department; (9) children’s hospital; (10) women’s health care; and (11) musculoskeletal and sports medicine.

### Sampling Technique

A sample size of 100 patients was used to detect methodological considerations to inform the design of the main study [19]. To ensure that the sample reflected the patient population at the study site, we first determined how many records to draw from each clinical service. This was done by allocating the sample in proportion to the number of admissions that each service received during the study period. For example, a total of 53,307 patients were admitted to the study site in 2019. Of these 53,307 patients, 10,543 (19.8%) were admitted to the internal medicine service. To calculate the number of internal medicine patients included in the total sample, we divided the number of patients admitted to internal medicine (ie, 10,543) by the total number of patients admitted to the study site (ie, 53,307) and multiplied the result by our total sample size (ie, 100). Therefore, 20 patients admitted to the internal medicine service were included in the total sample.

Next, for each service, a list of all admitted patients was prepared and sorted in order of admission date. To select a representative sample from each clinical service, we used systematic sampling. For every service, we calculated a sampling interval by dividing the total number of admissions in that service by the number of records needed from that service (this value is referred to as *K*). Next, we selected every *K*th patient from the ordered lists until the required number of records was reached. For example, to calculate the sampling interval (*K*) for internal medicine, we divided the total number of admissions to internal medicine (ie, 10,543) by the number of records needed from internal medicine for the total sample (ie, 20). Therefore, we selected every 527th patient admitted to internal medicine in 2019 to reach a total of 20 patients.

A total of 100 files were reviewed for tool 1A, and these patients are referred to as cohort A. However, 4% (4/100) of the patient files from cohort A were unavailable when tool 1B was applied, so they were replaced with 4 files from the same clinical services associated with those patients; these 100 patients are referred to as cohort B. In total, 104 patient files were reviewed.

### Definitions

We define an adverse event as “unintended physical injury resulting from or contributed to by medical care that requires additional monitoring, treatment, or hospitalization, or that results in death” [20].

### Data Collection

#### Overview

Our data collection approach used a 2-stage review process that is recommended by the World Health Organization for collecting data on adverse events using retrospective record reviews [21]. During the first stage, we assessed the medical records against a list of triggers present in the screening tools. The triggers used are specific occurrences, such as clinical outcomes, laboratory tests, and interventions, that are closely associated with adverse events and, therefore, aid reviewers in identifying iatrogenic harm. For this study, we used 2 separate screening tools: tool 1A and tool 1B. Each positive trigger was subsequently checked for its association with an adverse event. If an adverse event was confirmed, we performed a detailed review of the identified event using tool 2, the adverse event assessment tool. The tools were converted from paper-based instruments into electronic templates on the browser-based software REDCap (Research Electronic Data Capture; Vanderbilt University) so that they could be accessed on laptops, computers, and tablets [22].

Data were initially collected by a single reviewer (SH), a medical doctor by training, who had trained on 10 test cases before starting data collection. Each week, the collected data and identified potential adverse events were reviewed by 4 additional medical doctors, including the senior authors (F Asif and AL), who hold postgraduate qualifications and have extensive experience in patient safety and quality improvement. Consensus among all reviewers was required before any incident was designated as an adverse event to minimize bias. Furthermore, this iterative review process promoted consistency and progressive standardization across the study period.

#### Screening Tools (Tools 1A and 1B)

The screening tools are primarily made up of questions regarding patient characteristics and their corresponding trigger lists. The first trigger list, contained in tool 1A, was adapted from the work by Letaief et al [23]. The trigger list used in tool 1B was adapted from the Global Trigger Tool for Measuring Adverse Events developed by the Institute for Healthcare Improvement [24].

#### Adverse Event Assessment Tool (Tool 2)

Tool 2 was used to delineate the characteristics of the adverse events identified, including a confidence score for preventability. Tool 2 was also adapted from the work by Letaief et al [23]. A single patient file may contain evidence of multiple adverse events, and every adverse event had a corresponding tool 2 applied.

### Data Sources

The hospital uses physical patient files as well as electronic databases for information such as medication orders and laboratory test results to maintain detailed patient records. We used the physical files and all available electronic databases to conduct our study.

### Data Analysis

The main outcome measure was the rate of adverse events in the inpatient setting. The rate was reported as the number of adverse events per 100 hospital admissions. Furthermore, we reported the frequency of the different types of adverse events identified. Events with preventability scores above 3 (out of a maximum of 6) were classified as preventable. Data analysis was carried out using Stata (version 14; StataCorp).

### Ethical Considerations

This study was approved by the Ethics Review Committee at Aga Khan University (2023-6324-24566), covering secondary analysis of existing data without need for additional consent. We generated unique identifiers against which all findings were documented, and every effort was made to maintain the anonymity of the patients whose files were reviewed. Data were stored on encrypted, institution-approved cloud services that restricted access to only the investigating team.

### Patient and Public Involvement

Patients or the public were not involved in the design, conduct, reporting, or dissemination plans of this research.

### Reporting Guideline

We used the Strengthening the Reporting of Observational Studies in Epidemiology reporting guidelines to draft and edit this manuscript. The checklist has been included in Multimedia Appendix 1 [25].

## Results

The mean ages of patients in cohort A and cohort B were 39.2 (SD 27.7) and 39.8 (SD 28.4) years, respectively. Male individuals constituted 45% (45/100) of cohort A and 46% (46/100) of cohort B. The mean length of hospital stay for cohort A was 3.3 (SD 2.8) days compared to 3.5 (SD 3.4) days for cohort B. In cohort A, 36% (36/100) of the patients were admitted electively, whereas 39% (39/100) were admitted acutely. Conversely, in cohort B, 33% (33/100) of the patients were admitted electively, and 38% (38/100) were admitted acutely. Patient characteristics are summarized in Table 1.

**Table 1:** Comparison of demographics and tool performance (N=100).

				Tool 1A		Tool 1B
**Patient characteristics**						
	Age (y), mean (SD)			39.2 (27.7)		39.8 (28.4)
	**Sex, n (%)**					
		Female	55 (55)		54 (54)	
		Male	45 (45)		46 (46)	
	Hospital stay (d), mean (SD)			3.3 (2.8)		3.5 (3.4)
	**Admission status, n (%)**					
		Elective	36 (36)		33 (33)	
		Acute	39 (39)		38 (38)	
		Direct^a^	21 (21)		22 (22)	
		Did not know	4 (4)		7 (7)	
**Tool performance**						
	Time taken per file (min), median (IQR)			19 (13-27)		29 (19-57)
	Total number of positive triggers			114		260
	Number of patients with positive triggers			75		87
	Total number of adverse events			1		30
	Number of patients with adverse events			1		22

^a^The patient presented to an outpatient clinic and was directly admitted after the consultation.

A median time of 19 (IQR 13-27) minutes was required to review each file using tool 1A. A total of 114 positive triggers were found, resulting in the identification of 1 adverse event, which translates to an adverse event rate of 1 event per 100 hospital admissions. For tool 1B, a median time of 29 (IQR 19-57) minutes was required per file, and a total of 260 positive triggers were identified along with 30 adverse events, with 22% (22/100) of the patients experiencing at least one adverse event. Of these 30 events, 18 (60%) were considered preventable. The performance of both tools is compared in Table 1.

The most commonly identified trigger using tool 1A was >1 admission within 30 days, which was positive in a total of 27% (27/100) of the patients. Difference between diagnosis at admission and diagnosis at discharge was positive in 25% (25/100) of the patients, and National Early Warning Score 2 of ≥3 or Pediatric Early Warning Score of ≥2 on the day of discharge was positive in 21% (21/100) of the patients, representing the second and third most commonly identified triggers, respectively. The most frequently identified triggers in tool 1A are listed in Table 2.

**Table 2:** Most common positive triggers identified in cohort A using tool 1A (N=100).a

Trigger	Patients, n (%)
>1 admission within 30 days	27 (27.0)
Difference between diagnosis at admission and diagnosis at discharge	25 (25.0)
NEWS2^b^ of ≥3 or PEWS^c^ of ≥2 on the day of discharge (n=89)	21 (23.6)
>1 visit to the operating room within the last 6 months	13 (13.0)
Cardiac or respiratory arrest; low Apgar score	9 (9.0)
Injury or complications related to the termination of pregnancy or labor and delivery, including neonatal complications (n=32)	3 (9.4)
LAMA^d^	3 (3.0)
Unplanned transfer from general care to intensive care or higher dependency (n=84)	3 (3.6)
Development of neurological deficit not present on admission	2 (2.0)
Patient care delayed or lesser treatment given because the patient was unable to pay	2 (2.0)

^a^Some triggers were not applicable to all patients (eg, triggers for surgical care were not applicable to patients who did not undergo surgery).

^b^NEWS2: National Early Warning Score 2.

^c^PEWS: Pediatric Early Warning Score.

^d^LAMA: leave against medical advice.

Antiemetic administration, which occurred with 60% (60/100) of the patients, was the most frequently identified trigger using tool 1B, followed by transfusion of blood products, which was found in 19% (19/100) of the patients. The next most common trigger was time in the emergency department greater than 6 hours, which was found to be positive for a total of 18% (18/100) of the patients. The most commonly identified triggers using tool 1B are listed in Table 3.

**Table 3:** Most common positive triggers identified in cohort B using tool 1B (N=100).a

Trigger	Patients, n (%)
Antiemetic administration	60 (60.0)
Transfusion of blood products	19 (19.0)
Time in ED^b^ of >6 hours (n=39)	18 (46.2)
Oxytocic agent administration (n=16)	13 (81.3)
Administration of glucagon or glucose of ≥10% (n=20)	13 (65.0)
Vitamin K administration	13 (13.0)
Readmission within 30 days	12 (12.0)
Admission to intensive care postoperatively	11 (11.0)
Code, arrest, or rapid response call generated	11 (11.0)
Decrease of >25% in hemoglobin or hematocrits	9 (9.0)

^a^Some triggers were not applicable to all patients (eg, triggers for surgical care were not applicable to patients who did not undergo surgery).

^b^ED: emergency department.

Of a total of 30 events identified, surgical- or procedure-related complications were the most frequently identified type of adverse event, making up 40% (12/30) of all events identified. In this category, the most common event was surgery- or procedure-related hemorrhage, which was identified in 6% (6/100) of the patients and comprised 20% (6/30) of all events. Failure of diagnosis or treatment was the second most frequent category, making up 26.7% (8/30) of all events identified, followed by adverse medication events, which comprised 23.3% (7/30) of all events identified. The least common type of adverse event was health care–associated infections, only being identified in 1% (1/100) of the patients. Both tools 1A and 1B identified the health care–associated infection. The adverse events identified are summarized in Table 4.

**Table 4:** Categorization of all adverse events identified (N=30).

Category			Events, n (%)
**Surgery- or procedure-related complications**			12 (40.0)
	Hemorrhage	6 (20.0)	
	CNS^a^ complications	3 (10.0)	
	Iatrogenic injury	2 (6.7)	
	Postoperative atelectasis	1 (3.3)	
**Failure of diagnosis or treatment**			8 (26.7)
	Delay in diagnosis or treatment	5 (16.7)	
	Retinopathy of prematurity	2 (6.7)	
	Ventilator-associated injury	2 (6.7)	
	Failed extubation	1 (3.3)	
**Adverse medication event**			7 (23.3)
	Bleeding events	4 (13.3)	
	Drug-induced parkinsonism	1 (3.3)	
	Transfusion reaction	1 (3.3)	
	Oversedation	1 (3.3)	
Health care–associated infection			1 (3.3)^b^

^a^CNS: central nervous system.

^b^This adverse event was detected by both tool 1A and tool 1B.

## Discussion

This pilot study aimed to identify an appropriate methodology that could be used to accurately estimate the rate at which adverse events occur in hospitalized patients in LMICs. Using tool 1A, we obtained an adverse event rate of 1 event per 100 hospital admissions. However, using tool 1B, we obtained an adverse event rate of 30 events per 100 admissions, with a total of 22% (22/100) of the patients experiencing at least one adverse event. The most common type of adverse events identified were surgical- or procedure-related complications, with surgery- or procedure-related hemorrhage being the most frequently identified adverse event.

There is a lack of authoritative work regarding the incidence of adverse events in hospitalized patients in LMICs. However, the National Academies of Sciences, Engineering, and Medicine have estimated that 134 million adverse events occur across 531 million hospital admissions annually in LMICs, resulting in an adverse event rate of approximately 25 events per 100 hospital admissions [7]. However, the first methodology used, tool 1A, obtained an adverse event rate of 1 event per 100 hospital admissions. This is also in contrast to the work by Letaief et al [23], from which tool 1A was adapted. Their study found that, of 620 patient records reviewed, 62 (10%) contained evidence of at least one adverse event [23]. This suggests that tool 1A might be an inappropriate choice for estimating the rate of adverse events in our setting. We believe that this could be because the trigger list was originally developed and validated in HICs and, therefore, has limited applicability in our context. Differences in documentation practices, health care processes, and clinical workflows between HICs and LMICs may reduce the sensitivity and relevance of certain triggers. For example, variations in record-keeping systems, diagnostic resources available, and patterns of care delivery may lead to underdetection or misclassification of adverse events when using tools designed for high-resource settings [26].

The second methodology used, tool 1B, identified a total of 30 adverse events across 100 hospital admissions, which aligns more closely with regional estimates and, therefore, may be more suitable for use in our setting [7]. Moreover, Bates et al [27] conducted a similar study across 11 Boston hospitals using a trigger list that was also adapted from the Global Trigger Tool for Measuring Adverse Events. In their weighted sample of 2809 admissions, they identified at least one adverse event in 23.6% of the admissions, which is in line with our finding of 22% (22/100). This demonstrates that the tool may be applicable across diverse health care settings and is potentially suitable for future benchmarking efforts. It is worth noting that Bates et al [27] identified adverse medication events as the most common type of adverse event, accounting for 39% of all events detected. In contrast, surgical- or procedure-related complications were the most frequently identified type of adverse event in our study, comprising 40% (12/30) of all events recorded. We believe that this might reflect the difference between the quality of surgical care provided in LMICs compared with HICs [28]. Lack of access to timely surgical intervention, low surgical case volumes, and a lack of specialists are often cited as the main contributors to unsafe surgical care in LMICs [28].

An important consideration with tool 1B is that it generates a substantial number of positive triggers, many of which do not ultimately correspond to true adverse events yet still necessitate a detailed review of medical records. However, this broad approach may be preferable within nascent patient safety systems, especially in LMICs. By casting a wider net, tool 1B reduces the risk of missing relevant events and allows organizations to assess and prioritize identified events based on relevance and impact. Additionally, analyzing and addressing these triggers through a system-based approach may promote wider organizational improvements, yielding safety benefits and fostering resilience that may not be possible by relying solely on reactive approaches to safety issues [29].

We would like to acknowledge the salient limitations of this methodology. Retrospective chart reviews are limited by the fact that they only use data that were previously collected during the delivery of routine care and, therefore, cannot capture the full range of harm experienced by patients. For example, any adverse events that manifest after discharge but do not result in further patient encounters will be missed. Moreover, this issue is further exacerbated by poor documentation culture and practices in LMICs, which result in missing information, difficulty in interpreting information (such as due to the use of nonapproved abbreviations), and questionable authenticity of the information recorded. However, despite these limitations, retrospective chart reviews remain the most appropriate choice for our setting as they are relatively inexpensive; use existing data; and allow us to capture events that manifest after short latency periods, such as postoperative complications. Finally, we would like to acknowledge that tools 1A and 1B were not applied to the same set of medical records. However, as no adverse events were detected in the records involved, any potential impact on the findings is expected to be minimal.

In conclusion, this pilot study enabled us to identify and select well-defined, validated tools for use in our larger study, which will allow for reliable measurement of the incidence of adverse events among hospitalized patients. The methodological and operational lessons learned will directly inform our larger study and future capacity-building efforts in data quality, training, and surveillance systems [30]. Furthermore, by building local capacity for adverse event detection, we contribute to broader efforts to strengthen health systems by identifying priority areas for quality improvement, guiding resource allocation, and informing the design of context-appropriate interventions to reduce preventable harm. At the policy level, evidence generated through such locally led initiatives can support the development of national patient safety strategies, establish frameworks for data-driven decision-making, and integrate patient safety indicators into routine health system performance monitoring.

## Appendix

Multimedia Appendix 1 STROBE checklist.

## Abbreviations

HIC: high-income country
LMIC: low- or middle-income country
REDCap: Research Electronic Data Capture
STROBE: Strengthening the Reporting of Observational Studies in Epidemiology

## Data Availability

The datasets generated or analyzed during this study are available from the corresponding author on reasonable request.
